# First Global Consensus for Clinical Guidelines: Structured Recommendations (S2k‐Level Guideline Framework) for the Rehabilitation of the Edentulous Maxilla Based on Core Outcome Sets

**DOI:** 10.1111/clr.70084

**Published:** 2026-02-24

**Authors:** Frank Schwarz, Ronald E. Jung, Ina Kopp, Lisa Heitz‐Mayfield, Hom‐Lay Wang, The participants of the First Global Consensus Conference Group for Implant Dentistry (GCCG)

**Affiliations:** ^1^ Department of Oral Surgery, Implantology and Oral Medicine Goethe University Frankfurt am Main Germany; ^2^ Clinic of Reconstructive Dentistry, Center for Dental Medicine University of Zurich Zurich Switzerland; ^3^ AWMF‐Institute for Medical Knowledge Management, Philipps‐University Marburg Marburg Germany; ^4^ The University of Western Australia, International Research Collaborative, Oral Health and Equity, School of Human Anatomy and Biology Crawley West Australia Australia; ^5^ The University of Sydney, School of Dentistry, Faculty of Medicine and Health Sydney New South Wales Australia; ^6^ Department of Periodontics & Oral Medicine, University of Michigan School of Dentistry Ann Arbor Michigan USA

**Keywords:** consensus conference, core outcome set, edentulous maxilla

## Abstract

**Objectives:**

Rehabilitation of the edentulous maxilla poses major clinical and patient‐centered challenges. While implant survival and marginal bone level changes dominate the literature, patient‐reported outcomes (PROs) such as aesthetic satisfaction, comfort, and quality of life remain underrepresented. The Global Consensus for Clinical Guidelines (GCCG) aimed to develop an international, consensus‐based guideline for maxillary rehabilitation that integrates PROs and clinician‐reported outcomes (ClinROs) within a structured treatment workflow and to identify future research priorities.

**Materials and Methods:**

This S2k‐level guideline was developed through a multi‐phase process involving clinical experts, a methodologist, patients, and interdisciplinary contributors. Evidence synthesis included eight systematic reviews and six structured surveys. A Core Outcome Set (COS) of 34 critically important outcomes (10 PROs, 22 objective and 2 subjective ClinROs) was established via a three‐round Delphi process with 105 experts. Consensus recommendations were generated and voted upon by 105 delegates from 26 countries using nominal group technique and anonymous plenary voting.

**Results:**

The guideline defines 36 consensus‐based recommendations mapped to 34 COS outcomes, covering all phases of care, patient selection, diagnostics, surgical and prosthetic treatment, complication management, and maintenance. It emphasizes shared decision‐making, prosthetically driven planning, immediate protocols when feasible, and risk‐based maintenance to prevent surgical, biological, and technical complications. A clinical workflow checklist and decision trees support practical implementation.

**Conclusions:**

This is the first global guideline for edentulous maxilla rehabilitation integrating a COS across all treatment phases, fostering evidence‐based, outcome‐driven, and patient‐centered implant care worldwide.

## 1 Background and Scope

Maxillary edentulism markedly impairs oral function, speech, aesthetics, and psychosocial well‐being, resulting in a measurable decline in overall quality of life (Emami et al. 2013).

The rehabilitation of the edentulous maxilla remains a highly complex clinical scenario, requiring a careful balance between surgical feasibility, prosthetic design, and long‐term maintenance, all while accounting for local and systemic risk factors commonly associated with edentulous maxilla patients, especially among the elderly (Dawson et al. 2022; Herrera et al. 2022; Schwarz et al. 2021).

Another critical challenge in maxillary rehabilitation is the potential mismatch between clinician and patient priorities. In fact, clinician‐reported outcomes (ClinROs), such as implant survival, peri‐implant bone level changes, and prosthetic complications, continue to dominate the contemporary literature on edentulous maxilla rehabilitation, while patient‐reported outcomes (PROs), including satisfaction with aesthetics, comfort, speech, and quality of life, remain inconsistently reported and poorly standardized (Francisco et al. 2026; Lin, Chen, et al. 2026; Pannuti et al. 2026; Park et al. 2026; Romito et al. 2026; Sabri et al. 2026; Saleh et al. 2026; Thoma et al. 2026).

Despite recent efforts to define core domains and harmonized measurement tools for clinical trials in implant dentistry (Tonetti et al. 2023), the lack of adequate reporting of PROs using validated questionnaires undermines the evidence base for patient‐centered care and impairs shared decision‐making, especially in complex scenarios where treatment choices can greatly impact daily life.

The Global Consensus for Clinical Guidelines (GCCG) was established to address the persistent disconnect between ClinROs and outcomes that are more central to patients' lived experience. Focused initially on the rehabilitation of the edentulous maxilla, the 1st GCCG introduced a transparent, globally inclusive framework that systematically integrates PROs and ClinROs into evidence‐based, patient‐oriented clinical workflows, covering the full treatment continuum from patient selection to long‐term maintenance.

## 2 Organization and Methodology

### 2.1 Organization

The GCCG was founded as a collaborative initiative guided by three leading organizations in implant dentistry: the European Association for Osseointegration (EAO), the International Team for Implantology (ITI), and the Osteology Foundation (OF). These core partners provided funding, resources, and expertise for project management.

A network of additional partner organizations, including the Chinese Stomatological Association (CSA), Japanese Society of Oral Implantology (JSOI), Korean Academy of Oral and Maxillofacial Implantology (KAOMI), Oral Reconstruction Foundation (ORF), Osseointegration Society of India (OSI), and the Brazilian Society of Periodontology (SOBRAPI) further enriched the initiative by contributing diverse regional expertise and strengthening its international dissemination. Two publishers (Wiley, Quintessence) support the publication and global dissemination of the proceedings. An organizational overview of the 1st GCCG is presented in Table 1.

**TABLE 1:** Organization and structure of the 1st GCCG.

Steering committee	Christer Dahlin, Ronald E. Jung, Jörg Neugebauer, Charlotte Stilwell
Scientific leaders	Frank Schwarz, Hom‐Lay Wang
Methodologist	Ina Kopp
Scientific Task Force	Bilal Al‐Nawas, Gil Alcoforado, Christer Dahlin, Nikos Donos, Joseph Fiorellini, German Gallucci, Ronald Jung, Ina Kopp, Jörg Neugebauer, Frank Schwarz, Charlotte Stilwell, Robert Vogel, Hom‐Lay Wang
Survey experts	Giulia Brunello, Guo‐Hao Lin, Todd Schoenbaum, Franz Strauss
Organization	Sophie Maupoil, Philippe Brégaint (EAO), Kati Benthaus (ITI), Ana Turco, Heike Fania (OF)
Dates of the preparatory meetings	17.06.2024 (Scientific Task Force—Online). 08.07.2024 (Scientific Task Force—Online). 22.07.2024 (Scientific Task Force—Online). 16.09.2024 (Scientific Task Force—Online). 08.10.2024 (Scientific Task Force—Online). 25.10.2024 (Scientific Task Force, on Site—EAO Congress in Milan). 26.11.2024 (Scientific Task Force—Online). 23.01.2025 (Scientific Task Force—Online). 03.03.2025 (Scientific Task Force—Online). 18.03.2025 (Scientific Task Force—Online). 26.03.2025 (all delegates—Online). 16.04.2025 (Scientific Task Force—Online). 27.05.2025 (kick off calls WG 1 and WG 2—Online). 28.05.2025 (kick‐off call WG 3—Online). 30.05.2025 (kick‐off call WG 4—Online)

Working Group 1: Number of implants/timing of implant placement and loading. Group Chairs: Gil Alcoforado and Nikos Donos. Working Group: Samir Abou‐Ayash, Gustavo Avila‐Ortiz, Joao Carames, Paolo Casentini, Tali Chackartchi, Vivianne Chappuis, Stephen Chen, Helena Francisco, Paul Fugazzotto, William Giannobile, Yoshiyuki Hagiwara, Adam Hamilton, Saso Ivanovski, Sergio Kahn, Joseph Kan, France Lambert, Robert Levine, Jose Manuel Navarro, Ethan Ng, Turker Ornekol, Claudio Pannuti, Michael Payer, Giuseppe Romito, Todd Schoenbaum, Manish Kumar Singh, Sejal Thacker
Working Group 2: Zygomatic, standard‐length and short implants. Group Chairs: German Gallucci and Jörg Neugebauer. Working Group: Giulia Brunello, Daniel Buser, Krzysztof Chmielewski, Ramesh Chowdhary, Luca Cordaro, Ryuji Hosokawa, Ole Jensen, Ui‐Won Jung, Ye Lin, Nikos Mardas, Aleksa Markovic, Nikos Mattheos, Eitan Mijirtsky, Iva Milinkovic, Leandro Nunes, Kevser Pala, Waldemar Polido, Mariano Sanz, Frank Schwarz, Anton Sculean, Andreas Stavropoulos, Franz Strauss, Takashi Sumi, Florian Thieringer, Daniel Thoma, Yuseung Yi
Working Group 3: Diagnostic imaging, augmentation techniques, management of complications. Group Chairs: Christer Dahlin and Joseph Fiorellini. Working Group: Tara Aghaloo, Kang‐Min Ahn, Bilal Al‐Nawas, Mauricio Araujo, James Chow, Marcelo Faveri, Tobias Fretwurst, Markus Hürzeler, Darnell Kaigler, Fouad Khoury, Sungtae Kim, Marcel Kunrath, Hongchang, Lai, Guo‐Hao (Alex) Lin, Michel Messora, Isabella Rocchietta, Robert Sader, Muhammad Saleh, Junyu Shi, Jamil Awad Shibli, Simon Storgard Jensen, Tiziano Testori, Istvan Urban, Hom‐Lay Wang, Yiqun Wu
Working Group 4: Conventional dentures, implant overdentures and implant‐supported fixed dental prostheses. Group Chairs: Charlotte Stilwell and Ronald E. Jung. Working Group: Florian Beuer, Giulia Brunello, Jae‐Kook Cha, Jeanette Chua Keng‐Ling, Donald Curtis, Karim El‐Kholy, Brian Goodacre, Lisa Heitz‐Mayfield, Mohit Kheur, Sunjai Kim, Hisamoto Kondo, Purnima Kumar, Alejandro Lanis, David Norre, Bjarni Pjetursson, Srinivasa Rao Bhasmey, Irena Sailer, Misi Si, Amerian Sones, Franz Strauss, Stefan Wolfart, Nicola Zitzmann

### 2.2 Methodology

This consensus‐based guideline (S2k‐level) was developed in accordance with the methodological framework of the Standing Guideline Commission of the German Association of the Scientific Medical Societies (AWMF) and following the five pillars of trustworthy guideline development (AWMF, G. A. O. T. S. M. S. and Commission, S. G n.d.). The process was coordinated by a Steering Committee (SC), a Scientific Task Force (STF), and an independent methodology consultant (I.K.).

**FIGURE 1:**
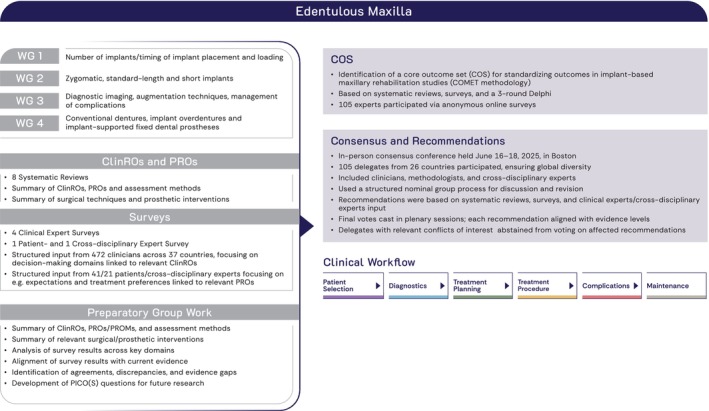
Methodological overview of the 1st GCCG. Evidence from eight systematic reviews and six structured surveys informed a three‐round Delphi process that established an indication‐specific Core Outcome Set (including critical PROs and ClinROs). Clinical recommendations informed by international experts, patients and cross‐disciplinary experts were developed and finalized at the consensus meeting employing nominal group technique and plenary voting.

The scientific foundation of the 1st GCCG was based on a comprehensive, multi‐layered framework. To ensure a focused and domain‐specific approach, the rehabilitation of the edentulous maxilla was thematically divided into four working groups (WGs), each addressing relevant clinical key domains: number of implants required, timing of implant placement and loading (WG1), zygomatic, standard‐length and short implants (WG2), diagnostic imaging, augmentation techniques, management of complications (WG3), and conventional dentures, implant overdentures and implant‐supported fixed dental prostheses (WG4) (Figure 1) (Donos et al. 2026; Fiorellini et al. 2026; Pala et al. 2026; Stilwell et al. 2026).

Each WG was chaired by internationally recognized experts and supported by a group of multidisciplinary delegates. Group Chairs, SC, and STF members held regular online meetings beginning in 2024 to ensure methodological consistency across groups. All delegates also participated in educational online sessions on guideline development principles and WG‐specific onboarding, supported by the methodology consultant.

### 2.3 Systematic Reviews on Clinician‐ and Patient‐Reported Outcomes

In a first step, eight systematic reviews were performed with the aim of identifying, categorising, and critically appraising the use and methodological quality of patient‐ (PRO) and clinician‐reported outcomes (ClinRO) documented in publications addressing the topics covered by the four WGs (Francisco et al. 2026; Lin, Chen, et al. 2026; Pannuti et al. 2026; Park et al. 2026; Romito et al. 2026; Sabri et al. 2026; Saleh et al. 2026; Thoma et al. 2026) (Table 2). The reviews adhered to PRISMA (Preferred Reporting Items for Systematic reviews and Meta‐Analyses) and COSMIN (Consensus‐based Standards for the selection of health status Measurement INstruments) methodologies and focused on studies from the past decade (years 2014–2024). They also summarized contemporary practices and adherence to standards in reporting outcomes related to surgical techniques and prosthetic interventions for the rehabilitation of the edentulous maxilla.

**TABLE 2:** Terminology of clinician‐ and patient‐reported outcomes in the GCCG Framework.

Outcome domain	Definition/examples (linked to COS)
PRO	Outcomes reported directly by the patient, reflecting their own perception of health, function, or quality of life, without interpretation by a clinician. These outcomes capture the subjective experience of treatment impact, including physical, functional, and psychosocial dimensions
PROM	Standardized, validated instruments (e.g., questionnaires, surveys, rating scales) used to quantify PROs in a reproducible and comparable manner. PROMs translate subjective patient experiences into structured data for clinical and research purposes
ClinRO assessment (objective)	Outcomes assessed and documented by a clinician that can be measured consistently and expressed in standardized, quantifiable terms. Within the GCCG framework, objective outcomes were considered those measurable with high consistency and minimal interpretive variability
ClinRO assessment (subjective)	Outcomes assessed by a clinician that involve interpretation or overall clinical judgment. The category of “subjective” outcomes was introduced pragmatically for the purposes of the GCCG initiative to reflect clinician‐perceived treatment success

### 2.4 Clinical Expert, Patient and Cross‐Disciplinary Expert Surveys

The SRs were complemented by six structured surveys, including four surveys of clinical experts linked to the individual WG topics, one survey targeting patients with lived experience and one survey including cross‐disciplinary experts. The findings of the surveys were used to identify outcomes relevant to patients and clinicians which had not been identified in the systematic reviews.

The clinical expert surveys gathered structured input from 472 clinicians across 63 countries, focusing on decision‐making domains such as diagnostics, implant number and configuration, grafting strategies, loading protocols, prosthetic design, and long‐term maintenance, each linked to relevant ClinROs (Brunello, Strauss, et al. 2026; Lin, Strauss, et al. 2026; Schoenbaum et al. 2026; Strauss et al. 2026).

The patient survey and cross‐disciplinary expert survey gathered structured input with a focus on expectations, treatment preferences, perceived burdens, and outcome priorities, also with particular emphasis on PROs, such as aesthetics, comfort, chewing function, speech, and psychosocial well‐being (Lin, Brunello, et al. 2026).

### 2.5 Preparation of the Consensus Conference in Working Group Meetings

In preparation for the in‐person consensus conference, online meetings for each WG together with the lead authors of the SRs were held to discuss the results of the SRs and the findings of the surveys. For each WG topic the following were summarised: (1) reported PROs and ClinROs identified in the SRs, (2) methods of assessment of the outcomes reported in the SRs, (3) description of all surgical/prosthetic interventions addressed in the reviewed literature, (4) survey results identifying additional outcomes and interventions for the clinical workflow domains: patient selection, diagnostics, treatment planning, treatment procedure, complications, and maintenance care, (5) alignment of the survey results with the current evidence (supporting and contradictory), for example, from recent SRs/meta‐analyses published within the previous 3 years (2021–2024); agreements and discrepancies with survey results were identified, and the corresponding references were provided, (6) survey results that could not be compared with current evidence due to a lack of relevant literature on the topic. These research gaps were translated into specific PICO(S) questions for future research. The resulting working documents served as a basis for the in‐person consensus conference (Figure 1). In addition, each WG was free to incorporate additional items deemed essential for accurately describing the clinical workflow, even if these were not covered by the literature identified in the SRs or the clinical expert surveys.

### 2.6 Core Outcome Set

A Core Outcome Set (COS) specific to the rehabilitation of the edentulous maxilla was developed through a structured, multi‐stakeholder process aligned with COMET (Core Outcome Measures in Effectiveness Trials) initiative standards. In this process, all outcomes identified in the SRs, clinical expert and patient/cross‐disciplinary expert surveys were rated for relevance by a three‐round Delphi process involving 105 international experts.

The Delphi process consisted of sequential, anonymous online questionnaires administered via SurveyMonkey (San Mateo, CA, USA), with links distributed by the EAO Office before and during the consensus meeting. As part of their responsibilities, all 105 GCCG delegates were invited to complete the survey. Information on the COS principles and consensus process was provided during the preparatory online sessions of the WGs as well as during the in‐person consensus conference to ensure all delegates were familiar with the Delphi process.

For the Delphi process, outcomes were evaluated using a standardized 9‐point Likert scale (i.e., Rounds 1 and 2) to identify their relevance from not important to critically important. During Round 3, consensus was reached on the outcomes considered critically important at various stages of the clinical workflow. Outcomes were subsequently categorized as 10 PROs, 22 objective (obj), and 2 subjective (subj) ClinROs.

Only outcomes rated as critically important (scores of 7–9 by ≥ 75% of respondents) were retained for formulating recommendations in this guideline. Each selected outcome was mapped to specific stages of care, ensuring consistency, clinical, and patient‐centered relevance across the entire clinical workflow (Brunello, Lin, et al. 2026).

Within this framework (PRO/PROM; ClinRO assessment—objective/subjective), objective outcomes were considered those measurable with high consistency and minimal interpretive variability, such as implant survival, width of keratinized mucosa, or marginal bone loss. The category of “subjective” outcomes, however, was introduced pragmatically for the purposes of the GCCG initiative to reflect clinician‐perceived treatment success, while recognizing that this classification has not been formally defined in the COMET Handbook or related COS literature (Williamson et al. 2017).

### 2.7 Consensus Process and Clinical Recommendations

**FIGURE 2:**
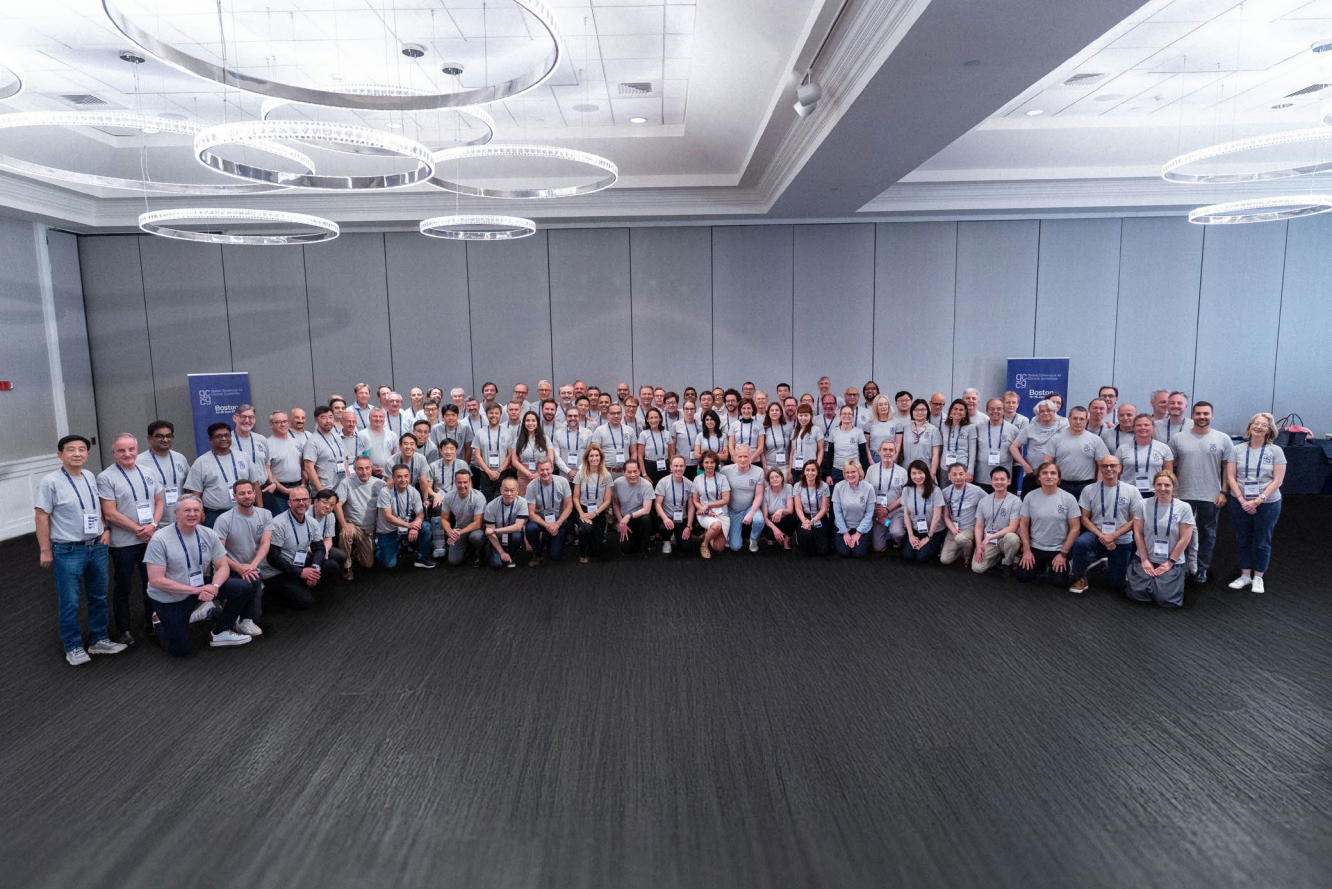
Group photo of participants in the 1st Global Consensus for Clinical Guidelines (GCCG) on the rehabilitation of the edentulous maxilla. Shown are members of the Steering Committee (SC), Scientific Task Force (STF), Working Group Chairs, international delegates, cross‐disciplinary representatives, and organizational staff, gathered during the in‐person consensus conference (Boston, MA, USA, June 2025).

The in‐person consensus conference (June 16–18, 2025, Boston, MA, USA) brought together 105 delegates from 26 countries. This ensured a broad geographical, cultural, and clinical diversity (Figure 2).

The Structured Consensus Conference (Murphy et al. 1998) was conducted over three days under the facilitation of an independent guideline methodologist.

On Day 1, participants worked within four topic‐related WGs, each facilitated by two Chairs and a Secretary with methodological support. After reviewing the survey and Delphi results and considering the available evidence, the groups engaged in structured discussions to draft recommendations and formulate PICO(S) questions for further research, applying the Nominal Group Technique.

On Day 2, the discussions shifted to a plenary session led by the independent guideline methodologist. WG Chairs presented their draft recommendations to the full assembly, where participants could raise questions, propose justified amendments, and debate unresolved issues. Suggested modifications were collected and consolidated by I.K. and the group chairs before being presented again to the plenum for preliminary voting. If consensus was not reached, further debate was encouraged, leading either to a final vote on revised proposals or referral back to the WGs for additional elaboration.

Day 3 combined further WG meetings to address tasks and refine recommendations with a second plenary session, where formal voting occurred for all recommendations.

The voting in the plenary sessions was conducted anonymously using the electronic system slido (https://www.slido.com/). PICO(S) questions suggested by WGs were not presented for formal voting but were presented by the WG Chairs and informally approved by the plenum.

Delegates could choose from the following voting options: agree, disagree, abstain, or abstain due to a conflict of interest. The outcomes were documented after each vote.

The Strength of Consensus was defined as follows:

**Table:** 

Strength of consensus	% Agreement
Strong consensus	Agreement of > 95% of delegates
Consensus	Agreement of 75%–95% of delegates
No consensus	Agreement of < 75% of delegates

### 2.8 Declaration of Interests and Potential Conflicts

All delegates (Table 1) disclosed secondary interests using the standardized ICMJE disclosure form (Editors, I. C. o. M. J 2012). Potential conflicts of interest (CoIs) were actively managed in accordance with Guidelines International Network (GIN) principles (Schunemann et al. 2015), both during online WG meetings and in plenary sessions. Delegates with relevant CoIs abstained from voting on affected recommendations, and abstention rates were transparently recorded. In cases where consensus was not reached, divergent viewpoints were documented and revisited in subsequent discussions to ensure balanced representation of perspectives.

## 3 Synthesis of the Recommendations

The rehabilitation of the edentulous maxilla requires a comprehensive, structured clinical workflow that guides the clinician from patient selection to long‐term follow‐up. This section briefly synthesizes the final 36 recommendations from all four WGs for the defined clinical workflow domains (Donos et al. 2026; Fiorellini et al. 2026; Pala et al. 2026; Stilwell et al. 2026).

**FIGURE 3:**
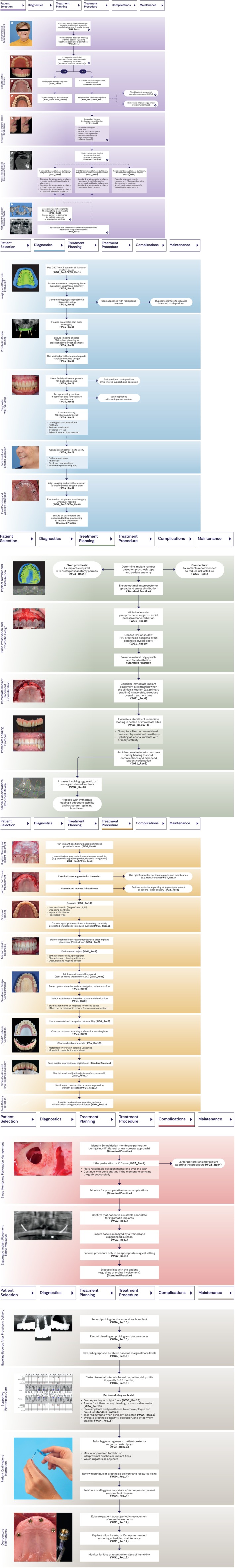
Decision‐trees for the rehabilitation of the edentulous maxilla based on simplified recommendations of WGs 1–4.

**FIGURE 4:**
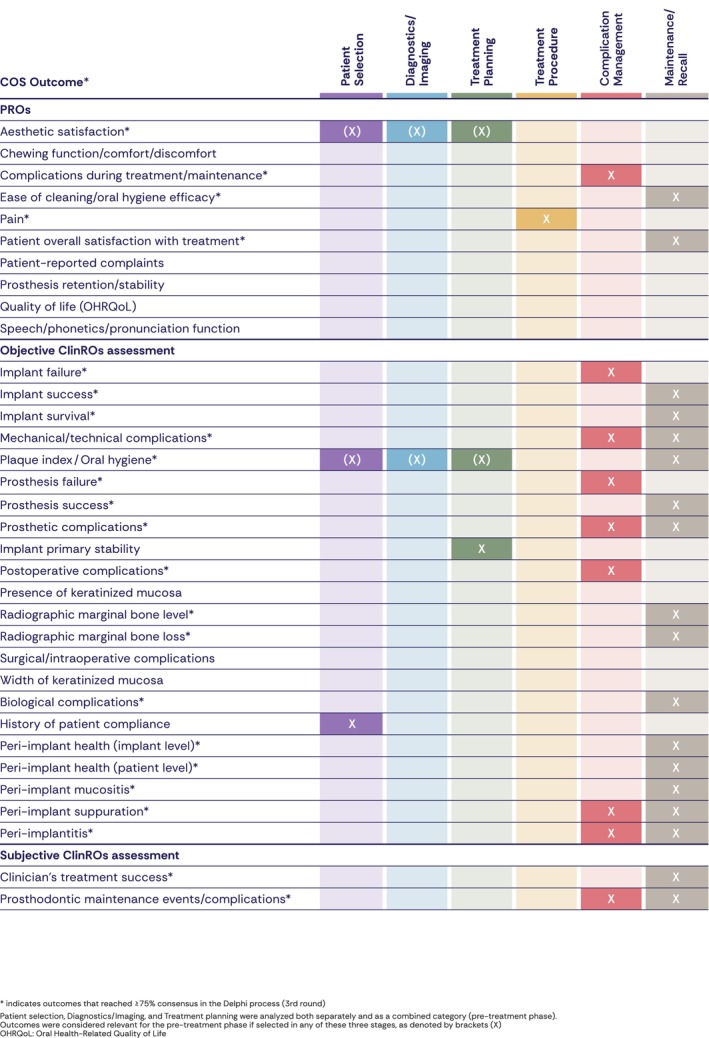
Core Outcome Set mapped to clinical workflow stages based on the Delphi process.

**FIGURE 5:**
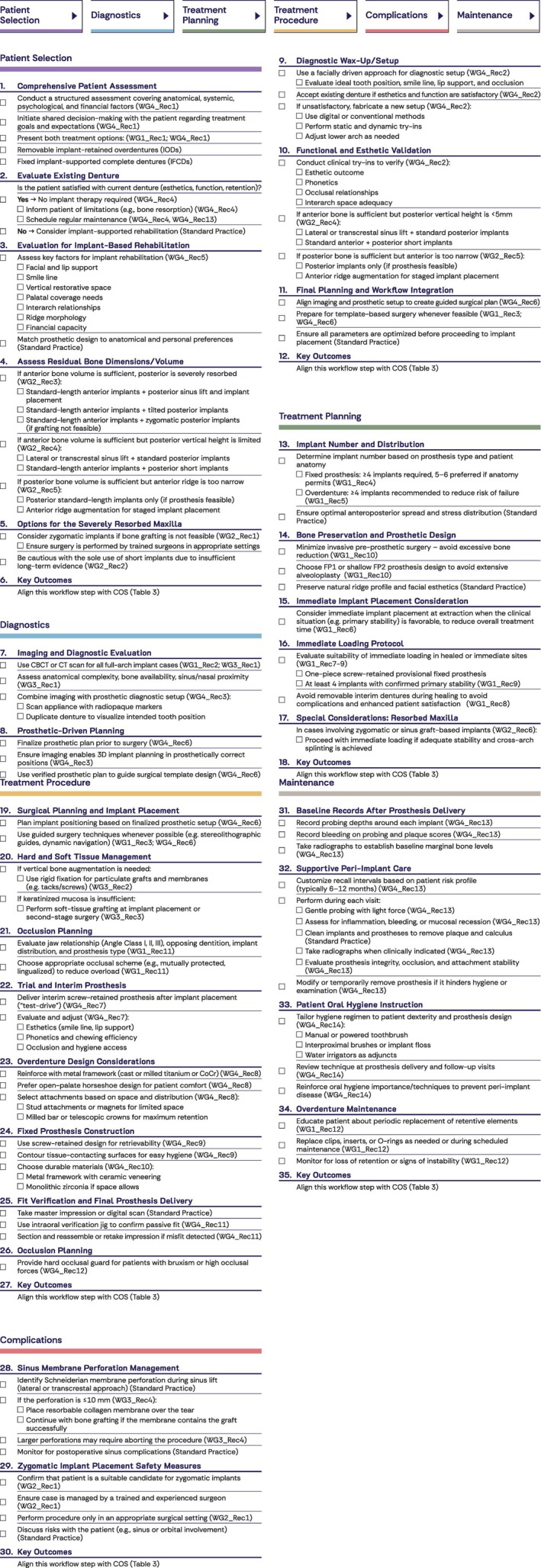
Checklists synthesizing the simplified recommendations of WGs 1–4 to clinical workflow stages.

Decision‐trees (Figure 3), COS (Figure 4), and checklists (Figure 5) are presented to summarize the main steps for day‐to‐day clinical practice.

### 3.1 Patient Selection and Treatment Decision‐Making

Treatment planning in the edentulous maxilla should begin with a thorough patient assessment and shared decision‐making process. In particular, all patients should be informed of all available treatment options, including conventional dentures (CD), implant overdentures (IODs), and implant‐supported fixed complete dentures (IFCD; synonym: implant‐supported fixed dental prosthesis [IFDP]), to allow for an informed and preference‐driven choice (WG1_Recommendation [Rec] 1; WG1_Rec1). A structured assessment covering anatomical, systemic, psychological, and financial factors, along with a frank discussion of risks, benefits, and the long‐term maintenance requirements, is essential. This ensures that the patient fully understands the commitments of implant therapy and that any chosen treatment path aligns with their motivation and expectations (WG4_Rec1). Notably, while many edentulous patients favor a fixed prosthesis for greater comfort and function, others prefer the simpler cleaning and maintenance of a removable overdenture. Thus, the final decision should account for the patient's individual priorities.

Given the increasing number of patients receiving antiresorptive and/or antiangiogenic therapies, a thorough medication history and MRONJ (medication‐related osteonecrosis of the jaw) risk assessment should be part of the initial evaluation. Implant‐supported rehabilitation is not universally contraindicated; however, treatment planning should be individualized based on the indication, route, dose, and duration of therapy, and should include interprofessional communication with the prescribing physician, optimization of oral health, and explicit informed consent regarding MRONJ risk.

If a patient is satisfied with the aesthetics, function, and retention of their existing conventional denture and no clinical problems are identified, no further therapy is required beyond regular maintenance. However, the patient should be informed of the long‐term limitations of denture use, such as ongoing bone resorption and reduced stability over time (WG4_Rec4). During the initial examination, the quality of the existing complete denture should be carefully evaluated to identify possibilities for its improvement or optimization, such as border extension, vertical dimension of occlusion, and the condition and function of the prosthetic teeth.

When an implant‐based rehabilitation is considered, clinicians should evaluate suitability based on, for example, facial and lip support, smile line, available restorative space with particular emphasis on the vertical dimension, palatal coverage requirements, interarch relationships, residual ridge morphology, and financial capacity (WG4_Rec5). These elements help determine the feasibility of treatment and guide the selection of an appropriate prosthetic design, for example, clarifying whether a fixed full‐arch denture or a removable overdenture would be more suitable given the patient's anatomy and expectations.

For the severely atrophic maxilla, where bone grafting is not feasible, zygomatic implants may be presented as an alternative to achieve a functional full‐arch prosthesis. Such cases must be handled by experienced surgeons in specialized surgical settings to manage the surgical complexity and avoid severe complications (WG2_Rec1). Short implants in the posterior maxilla are a potential option to reduce patient morbidity, but current evidence is insufficient to formally recommend their use as a standalone solution (i.e., a full‐arch rehabilitation solely supported by short implants) (WG2_Rec 2). Until the supporting evidence becomes more robust, clinicians should favor standard‐length implants or combined approaches in atrophic maxillae.

Additionally, treatment decisions should be guided by the patient's available residual bone volume:

- If the anterior maxilla has sufficient bone volume but the posterior maxilla is severely resorbed, consider: (1) standard‐length anterior implants with posterior bone augmentation/sinus lift and implant placement; (2) standard‐length implants placed in the available anterior bone with straight/tilted posterior implants placed in native bone if these allow for a significant improvement of anterio‐posterior spread and in prosthetically correct positions; or (3) standard‐length anterior implants combined with graftless options in the posterior (zygomatic implants) if grafting is not feasible and tilted implants do not offer any advantages (WG2_Rec3).
- If the anterior maxilla has sufficient bone volume, but the posterior regions have deficient vertical height but sufficient bone width, options may include: (1) standard‐length implants in the anterior and posterior in conjunction with bone augmentation/sinus lift (lateral or transcrestal); or (2) standard‐length anterior implants in combination with short implants in the posterior region (WG2_Rec4).
- If the posterior maxilla provides sufficient bone volume but the anterior ridge has an insufficient bone width, then either implants can be placed in the available posterior bone (straight or tilted) if the anterior–posterior spread is compatible with the prosthetic solution, or bone augmentation of the anterior ridge can be performed to enable staged implant placement of standard‐length implants (WG2_Rec 5).

In all scenarios, decision‐making should be personalized to the patient's anatomy, morbidity, and clinical needs, with the aim of considering relevant key COS outcomes (Brunello, Lin, et al. 2026) (Figure 3—Patient Selection).

### 3.2 Diagnostics and Prosthetic Planning

Accurate diagnostics are a key component in the clinical workflow. Three‐dimensional (3D) imaging, preferably cone‐beam computed tomography (CBCT) or CT scans, are recommended in all patients undergoing full‐arch implant therapy to thoroughly assess the complexity of the maxillary anatomy, identify any structural limitations, and enable safe, prosthetically driven planning (WG1_Rec2; WG3_Rec1). The recommendations encourage the routine use of CBCT in this complex clinical scenario, as it allows for accurate visualization of the anatomical structures, thereby facilitating the ideal positioning of implants and reducing risks for surgical complications. Whenever possible, the imaging process should be combined with a prosthetic diagnostic setup (such as a scan appliance with radiopaque markers or a duplicated denture) to visualize the intended tooth position in relation to the available bone (WG4_Rec3). The diagnostic setup or existing denture with markers is scanned using intraoral or lab scanners, and the scan is imported into the implant planning software. This prosthetically driven imaging approach ensures that implants are planned in the correct 3D positions required for the final restoration.

A facially‐driven prosthetic plan should be established before any surgical intervention. In practice, this should include a diagnostic wax‐up or trial denture (or a digital setup) to determine the ideal tooth positioning, smile line, lip support, and occlusal scheme for the patient (WG4_Rec 2). If the patient already has a well‐fitting maxillary denture with acceptable aesthetics and function, it can serve as a template. Otherwise, a new setup is indicated. Static and dynamic try‐in sessions are recommended to verify and refine the setup in the mouth, confirming proper aesthetics, phonetics, and jaw relations (WG4_Rec 2). Any necessary adjustments to the opposing dentition should also be considered.

This restoratively‐driven planning approach helps to avoid aesthetic or functional compromises at later stages of the treatment procedure, thereby reducing the risk of prosthetic complications (Figure 3—Diagnostics).

### 3.3 Treatment Planning

The number and distribution of implants required should be determined based on the planned prosthesis type, the patient's anatomy, and biomechanical considerations. For a fixed full‐arch implant‐supported prosthesis, a minimum of four implants is required, with five or six implants preferred when anatomy allows to improve support and stability as well as reduce the risk of overload or technical complications (WG1_Rec4). For a maxillary implant‐supported overdenture, using at least four implants is recommended to achieve sufficient retention and stability since two‐implant supported overdentures have shown higher failure and complication rates (WG1_Rec5).

For fixed full‐arch implant‐supported prostheses, implants should be distributed to maximize antero‐posterior spread and should be splinted in a rigid cross‐arch design. While four implants represent the minimum, the use of five to six implants (when anatomy and patient factors allow) is preferred to reduce cantilever length and distribute occlusal loads. Regarding framework design, a one‐piece cross‐arch framework is generally preferred; segmentation should not be routine and may be considered only in selected situations (e.g., extreme implant divergence or when needed to achieve passive fit and retrievability), while maintaining adequate rigidity and verifying passive fit (WG1_Rec4; WG4_Rec10–11).

Whenever feasible, clinicians should aim to preserve the existing bone and minimize invasive pre‐prosthetic surgery. If the patient's smile line and ridge volume permit, a fixed prosthesis (FP) replacing teeth only (FP1) or with minimal prosthetic gingiva (shallow FP2) is preferable, as it avoids the need for extensive alveolar bone reduction (alveoloplasty) and maintains the patient's natural bone and soft‐tissue profile (WG1_Rec10). This approach reduces surgical morbidity and preserves facial support.

In terms of treatment timeline, immediate protocols can significantly improve the patient experience and should be considered when clinical conditions allow. Immediate implant placement at the time of tooth extraction is a viable option if adequate primary stability is achieved and no acute infection or anatomical limitations are present (WG1_Rec 6). When implants are placed either immediately or at healed sites exhibiting good bone quality, immediate loading with a screw‐retained provisional full‐arch prosthesis is recommended whenever possible (WG1_Rec7–9). This approach can significantly shorten the overall treatment time and improve early patient satisfaction by avoiding any period without teeth or the use of a removable denture during healing. Also, in cases involving the use of zygomatic implants for a severely resorbed maxilla, if adequate primary implant stability and cross‐arch stabilization can be achieved, immediate loading with a provisional fixed prosthesis may be considered (WG2_Rec6). By splinting at least four implants in a cross‐arch interim prosthesis, the risk of micromovement is minimized (Figure 3—Treatment Planning).

### 3.4 Treatment Procedure (Surgical and Prosthetic Considerations)

Implant positioning in the edentulous maxilla should be guided by the prosthetic plan to ensure treatment success. Accordingly, and wherever feasible, it is suggested to use prosthetically‐driven guided surgery, either static (stereolithographic) guides or dynamic navigation, to translate the clinically verified, finalized diagnostic setup into prosthetically‐oriented 3D implant positions. Freehand placement remains appropriate where guided workflows are not feasible or offer no added value (WG1_Rec3; WG4_Rec6).

Adequate hard‐ and soft‐tissue management during surgery also contributes to long‐term success. In cases requiring vertical bone augmentation (e.g., onlay bone grafts or guided bone regeneration), any particulate graft material and barrier membrane should be rigidly fixed using either tacks or screws to prevent micromovement and early exposure (WG3_Rec2). Likewise, if the patient reveals an insufficient keratinized mucosa in the maxillary arch, soft‐tissue grafting (either performed at implant placement or at the second‐stage surgery) is recommended to create an adequate band of keratinized mucosa at the implant sites (WG3_Rec3).

In addition, occlusal considerations are also of crucial relevance. Therefore, the occlusion should be carefully planned based on the patient's jaw relationship (Angle Class I, II, or III), the opposing dentition (whether natural teeth or a prosthesis), the implant distribution, and the prosthesis type so that an appropriate occlusal scheme is implemented to minimize off‐axis forces (WG1_Rec11).

For full‐arch fixed cases, it is advisable to use a trial provisional prosthesis (“test‐drive”) before fabricating the definitive restoration. An interim resin prosthesis (often screw‐retained) delivered after implant placement is recommended to be worn for a trial period. This allows both the patient and clinician to assess aesthetics (smile line, lip support), function (chewing efficiency and phonetics), occlusion, and hygiene access, and to identify any necessary modifications (WG4_Rec7). On this basis, adjustments can be made in the final prosthesis to address issues noted during the trial phase.

For implant‐supported overdentures, the definitive prosthesis should be reinforced and designed for patient comfort. A metal framework (typically cast or milled titanium or cobalt‐chrome) within the acrylic denture base is recommended to prevent fractures, and a horseshoe (open‐palate) design may be preferred to improve the patient's taste perception and comfort (WG4_Rec8). The attachment system for an overdenture should be selected based on the number and distribution of implants and the available interarch space. For example, resilient stud attachments or magnets may be used when space is limited or when a simpler design is indicated, whereas a milled bar or telescopic crown system can provide maximum stability and retention if sufficient space and parallelism are available (WG4_Rec8).

For fixed full‐arch prostheses, a screw‐retained design is strongly recommended over cementation. Screw retention allows for easy removal of the prosthesis for maintenance or repairs and avoids the risk of submucosal excess cement around implants (WG4_Rec9). The contours of the prosthesis (particularly the tissue‐contacting surfaces and embrasures) should be shaped to facilitate the patient's self‐performed oral hygiene, ensuring that the patient can effectively clean under and around the restorations (WG4_Rec9).

To minimize technical complications, the definitive prosthesis should be made of durable materials. It is recommended to use a metal‐based framework (titanium or cobalt–chromium) with layered ceramic, or a monolithic milled zirconia arch prosthesis, depending on the implant configuration and available restorative space, with minimal cantilever (WG4_Rec10). These material/design choices have demonstrated good long‐term success and help reduce the incidence of prosthetic fractures or chipping.

When master impressions are made, it is critical to verify the accuracy of the working model or digital scan before fabricating the final prosthesis. An intraoral verification jig (splinting impression copings or abutments together in the mouth) should be used to confirm a passive fit of the multiunit framework on the implants (WG4_Rec11). If any misfit is detected, the index should be sectioned and reassembled, or a new impression should be taken to correct the discrepancy before proceeding to the definitive restoration. This extra step prevents undetected framework misfits that could otherwise lead to screw loosening, prosthesis instability, or concentrated stresses on the implants and marginal bone.

Finally, to protect the restoration after delivery, patients with bruxism or heavy occlusal forces should be provided with a protective night guard. A hard acrylic occlusal guard worn during sleep will help absorb excessive forces and is recommended to reduce the incidence of implant‐prosthesis fractures, screw loosening, or other complications over time (WG4Rec 12) (Figure 3—Treatment Procedure).

### 3.5 Management of Surgical Complications

While a variety of intraoperative complications can occur during edentulous maxillary implant surgery, this consensus focused on those deemed most pertinent. In particular, Schneiderian membrane perforation during sinus augmentation was identified as one of the most frequent complications requiring management (WG3_Rec4). If the sinus membrane is perforated during a lateral wall sinus lift, the recommendation is to carefully place a resorbable collagen membrane over the tear (for perforations up to approximately 10 mm) to seal the defect and then proceed with the bone graft if the membrane can effectively contain it. Using this method, many sinus perforations can be successfully managed without aborting the procedure, and it helps prevent postoperative sinus complications (WG3_Rec4). Although specific antibiotic regimens are beyond the scope of this guideline, clinicians may consider peri‐operative antibiotic coverage according to local protocols and patient risk profile, particularly when sinus augmentation is performed and/or when membrane perforation occurs, to reduce the risk of postoperative sinus infection and graft complications.

Zygomatic implant placement, as noted earlier, carries a risk of serious complications (such as sinus or orbital involvement) if not performed correctly. Critical risk control measures include strict case selection and limiting the procedure to appropriate surgical facilities and experienced and well‐trained surgeons who have received proper mentored clinical training, enabling them to conduct the treatment and manage potential severe complications (WG2_Rec1) (Figure 3—Complications).

### 3.6 Maintenance and Long‐Term Follow‐Up

Successful long‐term outcomes in maxillary implant rehabilitation depend on structured maintenance and patient adherence. At delivery of the final prosthesis, baseline peri‐implant parameters should be established (six‐point probing depths, bleeding on probing, and plaque indices) and intraoral radiographs made to define initial peri‐implant marginal bone levels.

WG4_Rec13 recommends recording baseline clinical parameters and intraoral radiographs at prosthesis delivery, followed by risk‐based recall and supportive peri‐implant care (SPIC) with regular monitoring of the peri‐implant health status including gentle peri‐implant probing, recording of bleeding/suppuration, plaque indices, and radiographs when indicated to ensure early detection of peri‐implant mucositis, suppuration, and peri‐implantitis (COS_objClinRO20–22). Follow‐up intervals should be individualized according to risk (typically every 6–12 months, with more frequent recalls for patients with a history of periodontal/peri‐implant disease).

Each maintenance visit should include a thorough evaluation of the implants and the implant‐supported/−retained prosthesis, including gentle peri‐implant probing with recording of probing depths and bleeding/suppuration, assessment of soft‐tissue health (inflammation, recession), professional debridement, and radiographs when clinically indicated (WG4_Rec13). The integrity of the prosthesis, occlusion, and (for overdentures) the stability of attachment components should be checked. If prosthesis design impedes hygiene or diagnosis (e.g., contours blocking access), temporary removal and/or design modification to improve access is suggested (WG4_Rec13).

Sustaining peri‐implant health also depends on effective home care. WG4_Rec14 emphasizes individualized oral‐hygiene instruction and reinforcement (e.g., twice‐daily brushing; interproximal brushes or specialty floss for under‐bridge/bar cleaning; water irrigators as adjuncts). Patients should demonstrate their technique at delivery and recalls so clinicians can adjust and reinforce instructions. These measures target oral‐hygiene efficacy (COS_PRO4) and reduction of plaque index scores (COS_objClinRO5).

For maxillary implant overdentures, patients should be informed about ongoing maintenance needs. WG1_Rec12 highlights timely replacement of retention components (e.g., clips/inserts/O‐rings) when retention declines or as deemed necessary to maintain function and prevent tissue trauma, thereby reducing prosthodontic maintenance events (COS_subjClinRO2).

In addition, durable materials and passive‐fit verification at the prosthetic stage help minimize mechanical/technical complications (COS_objClinRO4) and reduce maintenance needs (WG4_Rec10–11) (Figure 3—Maintenance).

## 4 Core Outcomes Linked to Checklist Recommendations by Workflow Stage

This section links the Core Outcome Set (COS) (Figure 4) with the final recommendations from Working Groups (WGs) 1–4 (Brunello, Lin, et al. 2026; Donos et al. 2026; Fiorellini et al. 2026; Pala et al. 2026; Stilwell et al. 2026). Each outcome is mapped to specific phases of the clinical workflow to illustrate how patient‐reported outcomes (PROs) and clinician‐reported outcomes (ClinROs) are interconnected through the already adopted recommendations.

For clarity, outcomes are referenced as COS_PRO (patient‐reported outcomes) and COS_objClinRO or COS_subjClinRO (objective or subjective clinician‐reported outcomes). The ≥ 75% stage‐specific consensus threshold refers to agreement on the primary workflow stage in which an outcome is assessed. Outcomes that did not reach this threshold may still be clinically relevant, as they often influence decision‐making across multiple workflow stages and are therefore addressed through corresponding WG recommendations.

### 4.1 Patient Selection and Treatment Decision‐Making

The COS identifies aesthetic satisfaction (COS_PRO1), functional comfort (COS_PRO2), and patient overall satisfaction with treatment (COS_PRO6) as key patient priorities during the initial planning phase. Accordingly, WG1 and WG4 emphasize shared decision‐making that compares conventional dentures, implant overdentures, and implant‐supported fixed prostheses, taking into account patient expectations, motivation, and long‐term maintenance requirements (WG1_Rec1; WG4_Rec1).

Assessment of patient compliance (COS_objClinRO17) is highlighted as a critical determinant of long‐term success and adherence (WG4_Rec1). A facially driven diagnostic setup is recommended to evaluate aesthetics, lip support, and function (WG4_Rec2), while CBCT imaging combined with radiopaque guides supports prosthetically driven implant planning (WG1_Rec2; WG3_Rec1; WG4_Rec3).

If a patient is satisfied with the aesthetics, function, and comfort of an existing conventional denture and no clinical problems are present, implant therapy may not be required (WG4_Rec4). This approach aligns treatment decisions with patient overall satisfaction (COS_PRO6) and quality of life (COS_PRO9) (Figure 4).

### 4.2 Diagnostics and Prosthetic Planning

In the Delphi mapping, no COS outcome reached the ≥ 75% threshold for exclusive assignment to the diagnostics and prosthetic planning stage. This reflects the fact that several outcomes influence, and are influenced by, multiple workflow phases rather than a single discrete step. Nevertheless, the following outcomes directly inform planning decisions through WG recommendations:

- Implant primary stability (COS_objClinRO9) guides the selection of immediate placement and loading protocols (WG1_Rec6–9; WG2_Rec6).
- Pain (COS_PRO5) and functional comfort (COS_PRO2) are indirectly affected by planning decisions that shorten treatment time and reduce the need for removable interim prostheses.
- Radiographic marginal bone level and marginal bone loss (COS_objClinRO12/13) support bone‐preserving strategies, including avoidance of excessive ridge reduction (WG1_Rec10).
- Mechanical and technical complications (COS_objClinRO4) and prosthesis retention/stability (COS_PRO8) inform occlusal planning and prosthetic design (WG1_Rec11).

WG2 provides decision‐making algorithms for managing the severely atrophic maxilla, including indications for zygomatic implants (WG2_Rec1–5). These planning decisions support implant success (COS_objClinRO2), implant survival (COS_objClinRO3), and prosthesis success (COS_objClinRO7) (Figure 4).

### 4.3 Treatment Procedure (Surgical and Prosthetic)

Several COS outcomes are primarily addressed during the treatment procedure stage, including pain (COS_PRO5), intraoperative complications and their management (COS_objClinRO10), and prosthesis success (COS_objClinRO7).

Immediate protocols play a central role at this stage. WG1_Rec9 recommends immediate loading with a one‐piece provisional screw‐retained fixed restoration, under favorable clinical conditions, to reduce implant failure (COS_objClinRO1) and complications during treatment and maintenance (COS_objClinRO10). In cases of severe maxillary atrophy, WG2_Rec6 allows immediate loading of zygomatic implants when adequate primary stability and cross‐arch stabilization are achieved.

Hard‐ and soft‐tissue management strategies (WG3_Rec2–3) aim to promote peri‐implant health at the implant level (COS_objClinRO18), improve oral hygiene efficacy (COS_PRO4), and address the width of keratinized mucosa (COS_objClinRO15). WG3_Rec4 provides guidance for managing Schneiderian membrane perforations during sinus augmentation.

Prosthetic try‐in with interim restorations (WG4_Rec7) enables assessment and refinement of aesthetic satisfaction (COS_PRO1), chewing function and comfort (COS_PRO3), and speech/phonetics (COS_PRO10). For implant overdentures, WG4_Rec8 supports open‐palate designs and metal reinforcement to enhance functional comfort (COS_PRO2) and reduce prosthesis failure (COS_objClinRO6).

WG4_Rec9–11 emphasize screw retention, passive fit, and hygiene‐friendly contours to minimize mechanical and technical complications (COS_objClinRO4) and support long‐term prosthesis success (COS_objClinRO7). For patients with parafunctional habits, WG4_Rec12 recommends occlusal guards to reduce prosthodontic maintenance events and complications (COS_subjClinRO2) (Figure 4).

### 4.4 Management of Complications

The complication management stage addresses outcomes that reached ≥ 75% consensus, including implant failure (COS_objClinRO1), prosthesis failure (COS_objClinRO6), biological complications (COS_objClinRO16), and peri‐implantitis (COS_objClinRO22).

WG2_Rec1–2 recommend limiting high‐complexity procedures, such as zygomatic implant placement, to experienced teams to reduce surgical and intraoperative complications (COS_objClinRO14). WG3_Rec4 outlines standardized approaches to managing sinus membrane perforations.

WG4_Rec13 emphasizes modification of prostheses that impair hygiene access, thereby supporting peri‐implant health at the patient level (COS_objClinRO19). Replacement of worn components and provision of occlusal guards (WG1_Rec12; WG4_Rec12) help preserve prosthesis retention and stability (COS_PRO8) and limit prosthodontic maintenance events and complications (COS_subjClinRO2) (Figure 4).

### 4.5 Maintenance and Long‐Term Follow‐Up

The maintenance phase encompasses the broadest range of high‐priority COS outcomes, including oral hygiene efficacy (COS_PRO4), plaque index/oral hygiene scores (COS_objClinRO5), radiographic marginal bone level and loss (COS_objClinRO12/13), implant success and survival (COS_objClinRO2/3), prosthesis success (COS_objClinRO7), mechanical and technical complications (COS_objClinRO4), biological complications (COS_objClinRO16), and peri‐implant diseases (COS_objClinRO20–22).

WG4_Rec13 recommends establishing baseline clinical and radiographic data at prosthesis delivery and monitoring these parameters over time. Long‐term maintenance should include routine peri‐implant probing at recall visits to enable early detection of peri‐implant mucositis, suppuration, and peri‐implantitis, thereby facilitating timely intervention.

Effective home‐care practices are essential to sustain peri‐implant health. WG4_Rec14 emphasizes individualized oral hygiene instruction and reinforcement, targeting oral hygiene efficacy (COS_PRO4) and reduction of plaque index scores (COS_objClinRO5). Regular follow‐up assessments (WG1_Rec12; WG4_Rec10–11) support early identification of prosthetic wear, prevention of mechanical and technical complications (COS_objClinRO4), and reduction of prosthodontic maintenance events and complications (COS_subjClinRO2).

Together, these measures contribute to long‐term implant success (COS_objClinRO2), prosthesis success (COS_objClinRO7), and peri‐implant health (COS_objClinRO18/19), while supporting clinician‐perceived treatment success (COS_subjClinRO1). Ultimately, structured maintenance care helps preserve patient overall satisfaction with treatment (COS_PRO6) and quality of life (COS_PRO9) following full‐arch rehabilitation (Figure 4).

## 5 Concluding Remarks

While the recommendations elaborated during the 1st GCCG were based on the best currently available evidence and broad international consensus, they are not intended to provide exhaustive procedural instructions for every clinical scenario or technique. Instead, the primary goal was to integrate and prioritize both PROs and ClinROs across a structured, globally applicable treatment workflow for the rehabilitation of the edentulous maxilla.

This guideline reflects a shift toward outcome‐driven, patient‐centered care, providing clinicians, researchers, and policy makers with a robust framework for shared decision‐making, individualized planning, and long‐term follow‐up. It offers a transparent methodology that aligns with the principles of trustworthy guideline development while respecting the diversity of clinical realities across regions, disciplines, and healthcare systems.

Clinicians are encouraged to apply the recommendations in the context of local expertise, infrastructure, and patient preferences, and to consult the broader literature, including SRs, randomized controlled trials, and professional education resources, for technical protocols, emerging innovations, and complementary evidence beyond the scope of this guideline.

Most importantly, the 1st GCCG represents a call to action: to routinely consider patient priorities alongside clinical evidence, to elevate the quality of outcome reporting, and to collaboratively advance standards of care in implant dentistry.

## Author Contributions

**Frank Schwarz:** conceptualization, funding acquisition, writing – original draft, methodology, writing – review and editing, formal analysis, project administration, validation. **Ronald E. Jung:** conceptualization, funding acquisition, writing – original draft, methodology, writing – review and editing, formal analysis, project administration, validation. **Ina Kopp:** formal analysis, writing – review and editing, validation, methodology. **Lisa Heitz‐Mayfield:** formal analysis, writing – review and editing, validation, methodology. **Hom‐Lay Wang:** writing – review and editing, writing – original draft, formal analysis, methodology, validation, conceptualization.

## Funding

The GCCG was funded by grants from the European Association for Osseointegration, the International Team for Implantology, and the Osteology Foundation. Publishing partners Wiley and Quintessence support the publication and global dissemination of the proceedings.

## Conflicts of Interest

All delegates disclosed secondary interests using the standardized International Committee of Medical Journal Editors (ICMJE) disclosure form. Potential conflicts of interest (CoIs) were actively managed in accordance with Guidelines International Network (GIN) principles.

## Data Availability

Data sharing not applicable to this article as no datasets were generated or analysed during the current study.
